# Analysis of the Measurement Properties of the Female Sexual Function Index 6-item Version (FSFI-6) in a Postpartum Brazilian Population

**DOI:** 10.1055/s-0043-1764496

**Published:** 2023-03-28

**Authors:** Julianna de Azevedo Guendler, Melania Maria Amorim, Maria Eduarda Melo Flamini, Alexandre Delgado, Andrea Lemos, Leila Katz

**Affiliations:** 1 Instituto de Medicina Integral Prof. Fernando Figueira, Recife, PE, Brazil; 2 Hospital das Clínicas de Pernambuco, Recife, PE, Brazil; 3 Universidade Federal de Pernambuco, Recife, PE, Brazil

**Keywords:** validation studies, questionnaire, sexuality, female, estudos de validação, questionário, sexualidade, feminino

## Abstract

**Objective** We evaluated internal consistency, test-retest reliability, and criterion validity of the Brazilian Portuguese version of the Female Sexual Function Index 6-item Version (FSFI-6) for postpartum women.

**Methods** Therefore, questionnaires were applied to 100 sexually active women in the postpartum period. The Cronbach α coefficient was used to evaluate the internal consistency. Test-retest reliability was analyzed by Kappa for each item of the questionnaire and by the Wilcoxon parametric test, comparing the total scores of each evaluation. For the assessment of criterion validity, the FSFI was used as the gold standard and the receiver operating characteristic (ROC) curve was constructed. Statistical analysis was performed using IBM SPSS Statistics for Windows, version 21.0 (IBM Corp., Armonk, NY, USA). It was found that the internal consistency of the FSFI-6 questionnaire was considerably high (0.839).

**Results** The test-retest reliability results were satisfactory. It can also be stated that the FSFI-6 questionnaire presented excellent discriminant validity (area under the curve [AUC] = 0.926). Women may be considered as having sexual dysfunction if the overall FSFI-6 score is < 21, with 85.5% sensitivity, 82.2% specificity, positive likelihood ratio of 4.81 and negative likelihood ratio of 0.18.

**Conclusion** We conclude that the Brazilian Portuguese version of FSFI-6 is valid for use in postpartum women.

## Resumo

**Objetivo** Avaliamos a consistência interna, a confiabilidade teste-reteste e a validade de critério do questionário FSFI-6 para a população brasileira no pós-parto.

**Métodos** Foram aplicados questionários em 100 mulheres sexualmente ativas no pós-parto. O coeficiente alfa de Cronbach foi utilizado para avaliar a consistência interna. A confiabilidade teste-reteste foi analisada pelo Kappa para cada item do questionário e pelo teste paramétrico de Wilcoxon, comparando-se os escores totais de cada avaliação. Para avaliar a validade de critério, o FSFI foi utilizado como padrão-ouro e a curva característica de operação de receptor (ROC, na sigla em inglês) foi construída. As análises estatísticas foram realizadas utilizando-se o software IBM SPSS Statistics for Windows, versão 21.0 (IBM Corp., Armonk, NY, USA).

**Resultados** Os resultados da confiabilidade teste-reteste foram satisfatórios. O FSFI-6 apresentou uma excelente validade discriminante (área sob a curva [AUC, na sigla em inglês] = 0,926). Considera-se presente a disfunção sexual se o escore geral do FSFI-6 for < 21, com sensibilidade de 85,5%, especificidade de 82,2%, razão de verossimilhança positiva de 4,81 e razão de verossimilhança negativa de 0,18.

**Conclusão** Concluímos que a versão em português do FSFI-6 se mostrou válida para uso em mulheres no pós-parto.

## Introduction

In the pregnancy and postpartum periods, numerous changes occur with women, including hormonal, anatomical, psychological, and social changes. Giving birth and becoming a mother is a source of many emotions and expectations. In general, there is a family restructuring that includes loss and/or decrease of the intimacy of the couple, to allow the reception of the newborn. Maintaining sexual activity after childbirth has been shown to be a key point in the quality of the relationship of the couple. 1 2

During the postpartum period, women may report at least one problem related to sexual function, including pain, decreased desire, difficulty in achieving orgasm, and vaginal dryness. 3 4 5 However, in Brazil, data such as those of prevalence in the postpartum period are still scarce. In a systematic review that included 20 studies that used validated questionnaires to identify the prevalence of sexual dysfunction in the Brazilian population, none of the included studies evaluated the postpartum population. 6

Questionnaires have been playing a large role in the evaluation of women with sexual dysfunction. They transform subjective measures into objective, quantifiable and analyzable data, in a global or specific way. Self-administered questionnaires are more typically used to assess female sexual function in clinical trials and epidemiological investigations. 7 8 In order for the questionnaire data to have credibility and validity, the measurement properties of the instrument should be evaluated. Credibility means that the instrument is capable of generating reproducible data or information. In the validation process, the measurement tool should be able to measure what it proposes to evaluate. 9

In 2009, a group of Italian researchers developed and validated an abbreviated form (6-item version) of the most popular psychometric diagnostic test currently used in the diagnosis of female sexual dysfunction, the Female Sexual Function Index (FSFI), to produce a faster screening test. 10 The use of the Female Sexual Function Index 6-item version (FSFI-6) was also proposed to facilitate the dialogue between the health professional and the patient and has already been validated for the general Korean, 11 Ecuadorian 12 and Portuguese 13 populations. However, there is no Brazilian version yet and the instrument has not been validated for use in postpartum women.

Thus, the present study aimed to evaluate the measurement properties (internal consistency, test-retest reliability, and criterion validity) of the FSFI-6 questionnaire in the Portuguese version for Brazil in postpartum women.

## Methods

This is a study of measurement properties performed through the application of the FSFI-6 questionnaire in Brazilian Portuguese to volunteers attended at the childcare outpatient clinic and the milk bank of the Instituto de Medicina Integral Prof. Fernando Figueira (IMIP) hospital in Recife, state of Pernambuco, Brazil. Data were collected between May and November of 2016. Inclusion criteria were: women in the postpartum period (up to 1 year postpartum); age ≥ 18 years old; and sexually active after delivery. Pregnant women and women diagnosed with cancer were excluded. The study was approved by the Research Ethics Committee in Human Subjects of the IMIP under the registration number 49172315.5.0000.5201. Before starting the evaluation, the women were invited to read and sign, if they agreed, an informed consent form. In addition, the authors of the original version of FSFI-6 in English authorized the translation and validation process. 10

The included sample considered the criteria of Mokkink et al., 9 which presupposes an adequate sample ≥ 100 for analysis of internal consistency, reliability, and criterion validity. Summing up, Sapnas et al. 14 state that “a minimum of 50 and a maximum of 100 subjects are sufficient when it is intended to evaluate the properties of instruments of health measures.” Therefore a final sample of 100 subjects was defined.

For data collection, three questionnaires were used. The first one was used to characterize the sample and was developed especially for this study, including sociodemographic data (age, body mass index [BMI], marital status, schooling, and occupation). The second and third instruments were the FSFI-6 and the FSFI, respectively. The first questionnaire was filled by a previously trained researcher and the others were self-administered.

The FSFI is a multidimensional questionnaire for the evaluation of female sexual function consisting of six domains: desire, arousal, lubrication, orgasm, satisfaction, and pain. For this, it consists of nineteen items that evaluate sexual function in the last 4 weeks and presents scores in each component. 15 The FSFI was originally developed in English, in 2008 had the transcultural adaptation to Brazil, 16 and later four other validation studies were conducted in different populations. 17 18 19 20 The FSFI was chosen as the gold standard for female sexual dysfunction evaluation, since it is widely used in studies of sexual dysfunction in different populations and because it is validated in several languages. 6 7

**Fig. 1:**
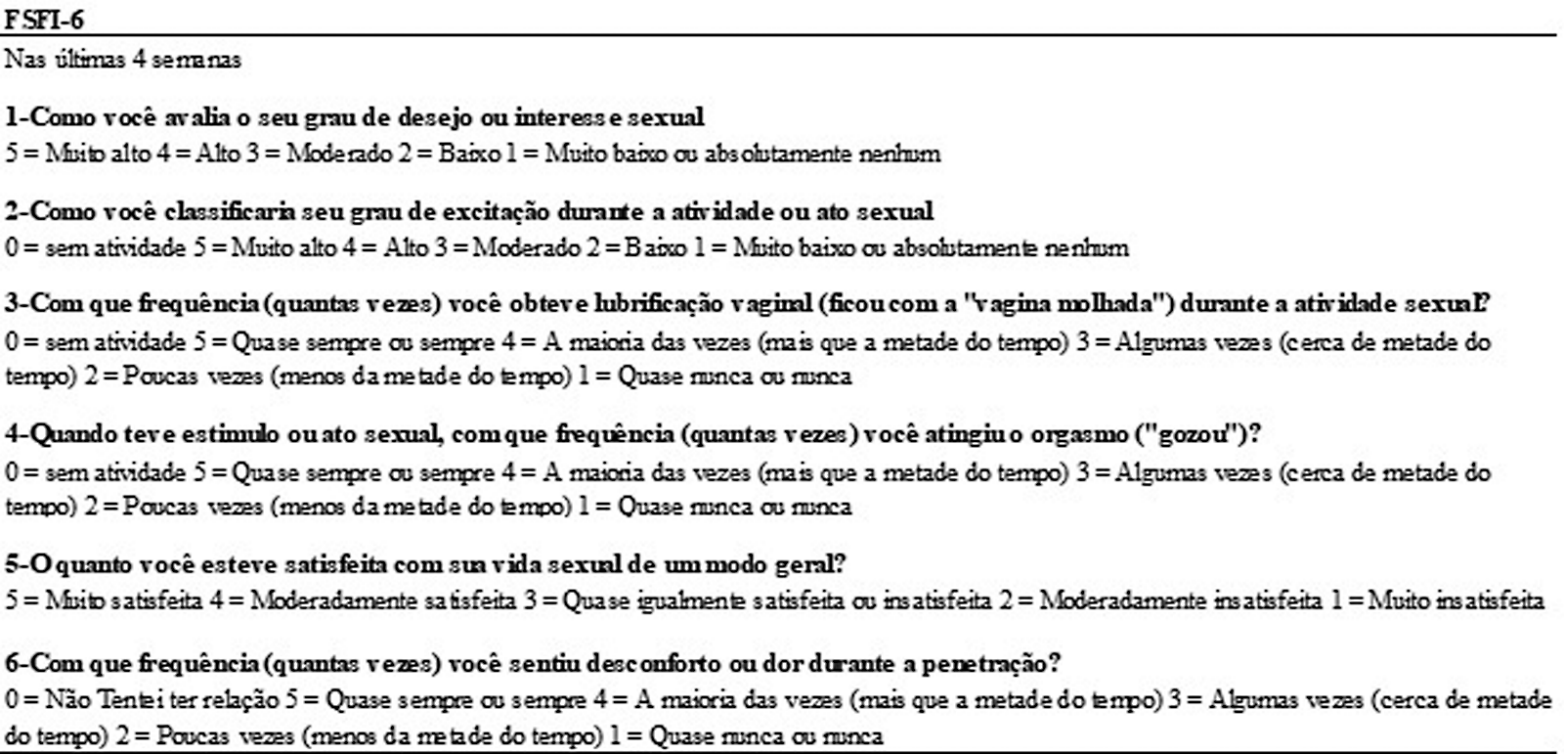
Components of the Portuguese version of the FSFI-6 questionnaire.

The study by Pacagnella et al., 16 which performed the transcultural adaptation of the FSFI to Brazil, involved five stages: translation, backtranslation, formal appreciation of semantic equivalence, final criticism by experts and pretest. As the original version of the FSFI-6 derived from the existing FSFI questions, the FSFI-6 questions were identified in the Brazilian version of the FSFI to set up the FSFI-6 Portuguese version ( Fig. 1 ) for the present study. One week after completing the questionnaires, the volunteers were asked to respond again to the FSFI-6, through scheduling, to perform the test-retest reliability analysis.

To characterize the sample, an exploratory descriptive analysis was performed. The qualitative variables were summarized in the form of absolute and relative frequencies (percentages), while the quantitative variables were summarized as measures of central tendency. We also evaluated the duration of the questionnaire application. To evaluate the internal consistency of the FSFI-6 questionnaire, the Cronbach α coefficient was used. This coefficient can be interpreted as a correlation coefficient that indicates an adjustment of the reliability of the scale, that is, it varies between - 1 and 1 and the higher this value, the better the reliability. Test-retest analysis was performed in two ways. In a first analysis, agreement was assessed through Kappa for each of the six questions in the FSFI-6 questionnaire. Kappa corresponds to a measure of agreement in which the value 0 indicates no agreement and the value 1 represents total agreement. Values of Kappa ≥ 0.7 are classified as sufficient by the Consensus-Based Standards for the Selection of Health Status Measurement Instruments (COSMIN) criteria. 21 The COSMIN criteria aim to improve the selection of outcome measurement instruments both in research and in clinical practice by developing methodology and practical tools for selecting the most suitable outcome measurement instrument. In the COSMIN taxonomy, there are three criteria: reliability, validity, and responsiveness. The reliability is: reliability (test-retest, inter-rater, intrarater), measurement error and internal consistency. The validity is: content validity, criterion validity, and construct validity. The responsiveness domain has only one domain that has the same name. 21

In the second analysis, the agreement between the test and the retest was analyzed through the Wilcoxon nonparametric test, comparing the total scores measured in the first and second evaluations. Criterion validity is the degree in which instrument scores are an adequate reflection of a gold standard. A good classification is given for criterion validity if the correlation with the gold standard is ≥ 0.7. 21 To investigate the criterion validity, a concurrent or discriminant analysis of the FSFI-6 questionnaire was performed compared with the FSFI, first calculating the scores of the six FSFI domains and the Spearman correlation coefficient between the six questions in the FSFI-6 questionnaire with the six FSFI domains. Subsequently, the receiver operating characteristic (ROC) curve of the FSFI-6 general score was calculated in relation to the classification of sexual dysfunction (FSFI score < 26.55) of the FSFI. Statistical analysis was performed using IBM SPSS Statistics for Windows, version 21.0 (IBM Corp., Armonk, NY, USA), considering a level of significance of 5%.

## Results

A total of 143 postpartum women were invited to the study, 8 of whom were excluded for being < 18 years old and 17 because of sexual inactivity. Of the eligible women, 18 refused to participate due to lack of time to respond to the questionnaires. The analyzed sample consisted of 100 postpartum women who answered the identification questionnaire and the FSFI and FSFI-6 questionnaires, without losses. According to the demographic characteristics of the women analyzed, the mean age was 26 years old (standard deviation [SD] 6.2), the BMI was 24.9 (SD 4.7), 43% had up to 6 months postpartum and 57% up to 1 year, 45% were grayish-brown, 62% were married or had a stable union, 47% had completed high school (12 years of study), and 67% were housewives. The application of the questionnaire lasted an average of three minutes. The prevalence of risk of sexual dysfunction among women was 55%, with scores < 26.55 being classified as sexual dysfunction with the FSFI questionnaire. Table 1 shows the result of the internal consistency analysis of the FSFI-6 questionnaire as measured by Cronbach α (0.839).

**Table 1:** FSFI-6 total result and by domains

Domains	Median (Q _1_ –Q _3_ )	Cronbach alpha
Q1- Desire	3.0 (3.0–4.0)	
Q2–Arousal	3.0 (3.0–4.0)	
Q3–Lubrification	3.0 (3.0–4.0)	
Q4–Orgasm	3.0 (2.0–4.0)	0.839
Q5–Satisfaction	4.0 (3.0–4.0)	
Q6–Pain	4.0 (3.0–5.0)	
Total Score	20.0 (18–23)	

Abbreviations: Q _1_ = 25 ^th^ percentile; Q _3_ = 75 ^th^ percentile.

Sample = 100 patients.

Regarding the agreement between the answers given in the first and second FSFI-6 evaluations (test-retest), in the first analysis, the calculated Kappa measures for each item were all above 0.7, with statistical significance ( Tables 2 and 3 ). The results of the Spearman correlation coefficient between the 6 questions in the FSFI-6 questionnaire with the 6 FSFI domains were: desire (0.75), lubrication (0.78), orgasm (0.76), satisfaction (0.73), arousal (0.81) and pain (0.94), as shown in Table 4 .

**Table 2:** Concordance analysis between the first and second evaluations of the FSFI-6, calculated by Kappa

*Item – FSFI-6*	Kappa ( *p-value* )
Q1	0.749 ( *p* < 0.001)
Q2	0.806 ( *p* < 0.001)
Q3	0.740 ( *p* < 0.001)
Q4	0.856 ( *p* < 0.001)
Q5	0.779 ( *p* < 0.001)
Q6	0.891 ( *p* < 0.001)

Sample = 100 patients.

**Table 3:** Comparison between the median scores of the first and second evaluations of the FSFI-6

	1 ^st^ evaluation	2 ^nd^ evaluation	Wilcoxon Test ( *p-value* )
FSFI-6–General Score Average (SD) Median (P _25_ –P _75_ )	19.80 (4.48) 20 (18–23)	19.83 (4.64) 20 (18–23)	0.461

Abbreviations: P25, 25th percentile; P75, 75th percentile; SD, standard deviation.

**Table 4:** Spearman correlation between the FSFI-6 and the FSFI domains ( *n* = 100)

FSFI	FSFI-6					
	Q1	Q2	Q3	Q4	Q5	Q6
Desire	0.757	0.548	0.410	0.387	0.434	0.480
Arousal	0.631	0.816	0.607	0.516	0.599	0.440
Lubrification	0.412	0.557	0.780	0.450	0.413	0.384
Orgasm	0.406	0.493	0.493	0.769	0.434	0.399
Satisfaction	0.385	0.559	0.443	0.523	0.738	0.348
Pain	0.380	0.361	0.332	0.417	0.283	0.949

All cases: *p* < 0.05.

**Fig. 2:**
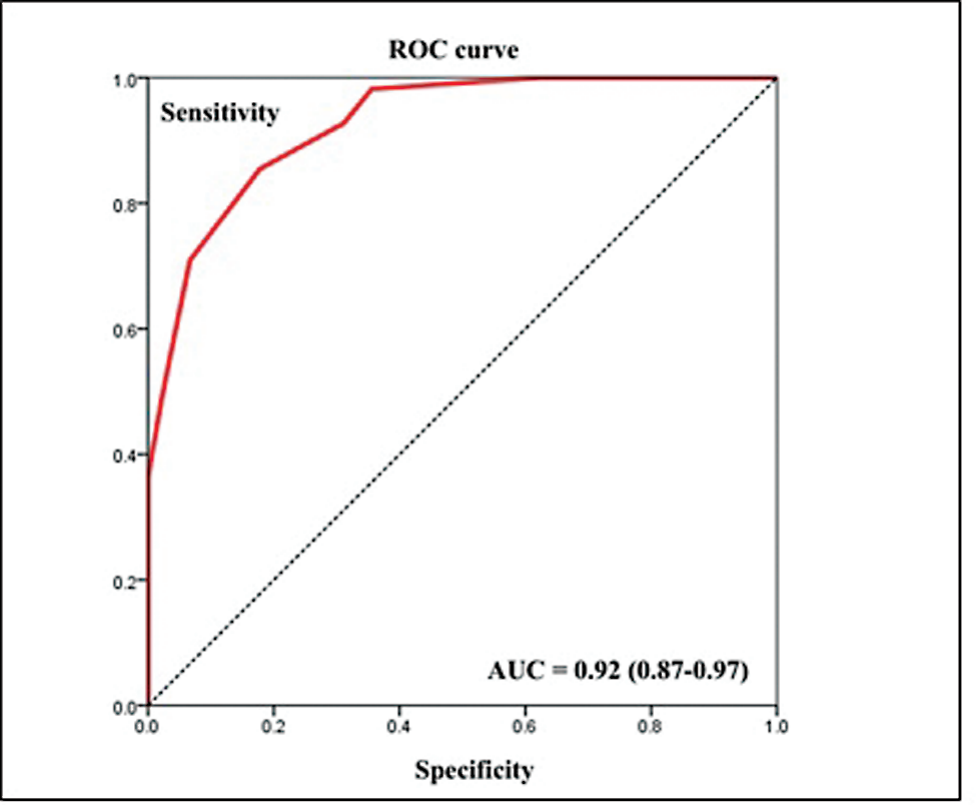
ROC curve of the FSFI-6 general score.

The ROC curve of the FSFI-6 general score in relation to the FSFI sexual dysfunction classification (FSFI score < 26.5) showed a discriminant validity area under the ROC curve [AUC] = 0.92 (0.87–0.97) ( Fig. 2 ). The diagnosis of sexual dysfunction can be considered if the general FSFI-6 score is < 21, with sensitivity of 85.5% (95% confidence interval [CI]: 0.73–0.92), and specificity of 82.2% (95%CI: 0.68–0.90). Positive and negative predictive values were 85.4% (95%CI: 0.78–0.92) and 82.2% (95%CI: 0.74–0.89), respectively, while the positive likelihood ratio was 4.81 (95%CI: 0.68–0.90) and the negative likelihood ratio was 0.18 (95%CI: 0.09–0.34).

## Discussion

The present study evaluated the measurement properties of the Brazilian Portuguese version of the FSFI-6 for Brazilian women in the postpartum period. To our knowledge, this is the first study to evaluate the measurement properties of a questionnaire on sexual dysfunction in postpartum women in Brazil and the results showed that FSFI-6, used in this population, had a fast application and presented good internal consistency, test-retest reliability, and discriminant validity.

A relevant data confirmed in the present study was the small time needed for the test application, in average 3 minutes. In the developmental study of the FSFI-6, while the mean time to respond to the original FSFI was 13 minutes, self-administration of the FSFI-6 took on average 1.5 minutes. 1

Less time to respond to the questionnaire is a major ally in the investigation of sexual dysfunction in the puerperium, because it facilitates dialogue between health professionals and patients on a frequently neglected topic, since only the minority of women have the initiative to talk about their difficulties and only a fraction of physicians questions the sexual function of their patients due to embarrassment or lack of knowledge of the sexual response. A brief instrument capable of pointing to sexual dysfunction in a few minutes allows more women to be investigated and those identified with dysfunction can be referred for further examination. In this way, it could be a useful tool, initially for epidemiological and clinical research, and potentially become a routine screening test.

Analyzing the results, we can infer that the reliability of the FSFI-6 questionnaire, evaluated by Cronbach α (0.83), was good. According to the COSMIN criteria, 21 Cronbach α must be between 0.7 and 0.95. The results were similar to those of the original version of the FSFI-6 (0.78) and the Korean (0.88) and Portuguese (0.80) versions, 11 13 with a good internal consistency in all versions of the questionnaire. The small variation of the values can be attributed to differences in the characteristics of the samples, since the coefficient is an inherent property of the response pattern of the studied population and may undergo changes depending on the population in which the scale is applied. 22

The test-retest reliability results were satisfactory. The interval of seven days between the applications of the instruments in the present study was within the recommended in the literature. According to Mokkink et al. 13 criteria, the interval between repeated administrations should be long enough to avoid remembrance, although short enough to ensure that clinical change has not occurred. Oftentimes, 1 week or 2 will be appropriate. In the evaluation of the Korean version, the follow-up for the retest was conducted 3 to 4 weeks after the first evaluation and obtained a median result. Changes in sexual activity between the events could explain the variations identified and justify the divergence in the results. 11

In terms of FSFI-6 criterion validity performed with the original FSFI and its dimensions, the strong correlations presented would be expected, since the questions of the two instruments overlap. However, this comparison was necessary to prove that the reduced version of the FSFI reflects the sexual dysfunction domains evaluated in the FSFI.

In the FSFI-6 development study, as there was no gold standard test to identify women with and without sexual dysfunction, sexological and physical examinations were performed, followed by the FSFI, and a 100% agreement was found between the result of the sexological examination and the FSFI. 1 Also based on the COSMIN criteria, 21 to evaluate criterion validity, the AUC should be ≥ 0.7 in correlation with the gold standard. The FSFI-6 questionnaire presented an excellent discriminant validity (AUC = 0.926), similar to the original version (AUC = 0.984) and to the Korean version (AUC = 0.948). 1 11

The ROC curve is a didactic way of representing the relationship between sensitivity and specificity. The greater the proximity of the curve to the upper left corner of the graph, the better the accuracy of the test, since the percentage of true positives approaches one and the percentage of false positives approaches zero. Through the ROC curve we can calculate the proportion of patients who were correctly classified by the test. In the present study, the cutoff point was defined as 21 points, because it was the score that allowed greater accuracy. This score is very close to that defined in the original, as well as in the Korean and Ecuadorian versions, which were ≤ 19, ≤ 21, and ≤ 20, respectively. 1 11 12

High results in positive and negative predictive values indicate the good performance of a test in the study population. If the test is positive and indicates sexual dysfunction, the positive predictive value of 85.4% found in the present study means that the individual has an 85.4% chance of actually having sexual dysfunction. If the test is negative and indicates no sexual dysfunction, a negative predictive value of 82.2% means that the individual has an 82.2% chance of actually not having sexual dysfunction.

Predictive values are influenced by the prevalence of the event in the studied population. Since this study had no population base, and was performed in a sample of outpatients, likelihood ratios became important information, since they were independent of the prevalence of the event studied. 23

In analyzing the likelihood ratio, we can say that it was very good. In other words, if the score is < 20.4, the probability of a woman having sexual dysfunction in the postpartum period increases 4.81, almost 5 times more, which would justify, on the vision of evidence-based health, the use of the questionnaire in the screening of sexual dysfunction in this population.

The choice of FSFI as the gold standard for the evaluation of sexual dysfunction was a limitation or bias of the present study, since the FSFI contains the same questions as the FSFI-6, and may have overestimated the ability of the test, consisting of a bias of incorporation. However, the FSFI was the chosen instrument due to the lack of other international tools validated in Brazil to identify sexual dysfunction and its frequent use in clinical research. In addition, it has been used as the gold standard in previous studies, such as the Korean and Portuguese validation studies. 11 13

The study could also have been more representative of the population if it could have been subdivided into a normative sample of convenience of the general population and a clinical sample of convenience of patients diagnosed with sexual disorders. However, with the topic of sexuality being still very little explored, a specific division for diagnosis and follow-up of sexual dysfunctions of puerperal women does not exist in the referral hospital studied. We followed the recommendation of the American College of Obstetricians and Gynecologists (ACOG) and classified the postpartum period as up to 1 year. 4

The implicit objective of our study was to provide a questionnaire for the evaluation of sexual dysfunction in postpartum women to contribute to an improvement in the described scenario of a lack of prevalence data and, mainly, to favor the dialogue between health professionals and women about sexuality. We have as a limitation the heterogeneity in relation to the postpartum period. There is inclusion of women in very different phases and realities, including about sexuality. However, the objective was to validate the FSFI-6 for postpartum women within 1 year, making it difficult to categorize the validation of the instrument by postpartum period.

As strengths of the study, we can list the use of the appropriate sample size for a study of measurement property, the performance of the retest within a time range recommended by the literature and, mostly, the availability of a quick tool, capable of stimulating clinical and epidemiological investigations of a topic of great importance to the quality of life of women.

## Conclusion

The results obtained with the present study show that the FSFI-6 in Brazilian Portuguese proved to be robust by the analysis of its measurement properties and its brevity. As perspectives for future research, it would be important to carry out cohort studies to monitor the sexual function of postpartum women and to allow the analysis of associated factors. It would still be relevant to evaluate responsiveness, a property to detect differences between two points in time (change over time) within groups, that is, a clinically relevant change in the scores of a measure of health-related functional status, so that the questionnaire can also be used to monitor the treatment of sexual dysfunction. Another possibility of research would be the comparison of the data obtained with the FSFI-6 with the findings of a physical evaluation of the pelvic floor.
